# Long QT syndrome with potassium voltage-gated channel subfamily H member 2 gene mutation mimicking refractory epilepsy: case report

**DOI:** 10.1186/s12883-021-02365-8

**Published:** 2021-09-04

**Authors:** Huicong Kang, Lili Lan, Yuchao Jia, Cun Li, Yongkang Fang, Suiqiang Zhu, Heidi Kirsch

**Affiliations:** 1 Department of Neurology, Tongji Hospital, Tongji Medical College, Huazhong University of Science and Technology, No. 1095 Jiefang Blvd., Wuhan, 430030 Hubei Province China; 2 Department of Neurology and Radiology & Biomedical Imaging, Epilepsy Center, University of California, San Francisco, California, 94143-0628 USA

**Keywords:** Long QT interval syndrome, Ventricular tachycardia, Torsades de pointes, *KCNH2*, Case report

## Abstract

**Background:**

Epileptic seizures can be difficult to distinguish from other etiologies that cause cerebral hypoxia, especially cardiac diseases. Long QT syndrome (LQTS), especially LQTS type 2 (LQT2), frequently masquerades as seizures because of the transient cerebral hypoxia caused by ventricular arrhythmia. The high rate of sudden death in LQTS highlights the importance of accurate and early diagnosis; correct diagnosis of LQTS also prevents inappropriate treatment with anti-epileptic drugs (AEDs).

**Case presentation:**

We report a case of congenital LQT2 with potassium voltage-gated channel subfamily H member 2 gene (*KCNH2*) mutation misdiagnosed as refractory epilepsy and treated with various AEDs for 22 years. The possibility of cardiac arrhythmia was suspected after the patient presented to the emergency room and the electrocardiograph (ECG) monitor showed paroxysmal ventricular tachycardia during attacks. Atypical seizure like attacks with prodromal uncomfortable chest sensation and palpitation, triggered by auditory stimulation, and typical ventricular tachycardia monitored by ECG raised suspicion for LQT2, which was confirmed by exome sequencing and epileptic seizure was ruled out by 24-h EEG monitoring. Although the patient rejected implantation of an implantable cardioverter defibrillator, β blocker was given and the syncope only attacked 1–2 per year when there was an incentive during the 5 years follow up.

**Conclusions:**

Our case illustrates how long LQTS can masquerade convincingly as epilepsy and can be treated wrongly with AEDs, putting the patient at high risk of sudden cardiac death. Careful ECG evaluation is recommend for both patients with first seizure and those with refractory epilepsy.

## Background

Long QT syndrome (LQTS), known as delayed repolarization syndrome, is a group of arrhythmogenic disorders affecting 1/2000 to 1/7000 people [1]; it is characterized by syncope and a high incidence of ventricular arrhythmia (including torsades de pointes) and sudden cardiac death. The mortality is greater than 20% in the year after the first syncopal event, nearly 50% at 5 years and 80% at 10 years [2]. Mortality is greatly reduced with early diagnosis and appropriate management. However, LQTS masquerading as epilepsy has been reported frequently because of similar clinical manifestations [3]; death due to LQTS may even mimic sudden unexplained death in epilepsy [4]. The possibility that similar channelopathies can underlie both LQTS and epilepsy in one patient was also reported [5, 6].

We present a patient with LQTS misdiagnosed as refractory epilepsy for 22 years, who tried multiple anti-epileptic drugs (AEDs) mainly based on her “epileptic” presentation. The patient has never been suspected LQTS because her normal routine 12 channel electrocardiograph (ECG) at early disease stage, until we caught clinical attacks during her initial visit to our emergency room (ER) and documented simultaneous cardiac arrhythmia. Diagnosis of LQTS type 2 (LQT2) was confirmed by ECG and the detection of potassium voltage-gated channel subfamily H member 2 gene (*KCNH2*) mutation and epilepsy was ruled out and all AEDs were withdrawn based on her normal 24-h video electroencephalograph (VEEG) monitoring.

## Case presentation

Medical history was obtained by interviews with the patient and family as well as a detailed review of outside medical records. The patient signed a written informed consent for publication of her case and any accompanying images at the same time.

She had head computed tomography (CT), 24-h VEEG, 12-channel dynamic ECG and blood analyses. Exome sequencing of cardiomyopathy and ion channelopathy was performed at the gene diagnosis center, Tongji Hospital affiliated to Tongji Medical College, Huazhong University of Science and Technology with methods previously reported [7].

A 35-year-old woman with a past medical history of chronic hepatitis B, and prematurity (born at 35 weeks; Apgar score 8) presented to the neurological ER after a syncopal event with cardiac arrest 2 h earlier, in the setting of sleep deprivation. She had a history of 22 years of paroxysmal generalized seizures with unconsciousness. Her first seizure at 13 years of age occurred while she was running a 1000 m race. The witness described that she suddenly stopped running and stared ahead, thrashed her limbs several seconds later, and collapsed with loss of consciousness, and then subsequently developed limb twitching, cyanosis of the face, and foaming at the mouth. About 2 min later, she stopped moving and had urinary incontinence. She recovered immediately and could not recall anything during the seizure, though she recalled running prior to the seizure. She was evaluated at the local hospital with normal glucose and electrolyte levels, normal head CT, and normal routine electroencephalograph (EEG) and 12-channel routine ECG and discharged without AED, as this was her first seizure. She had no risk factors for epilepsy, including no history of febrile convulsions, brain trauma or intracranial infection. Unfortunately, a similar seizure occurred 2 months later while she was shopping at supermarket. From then on, she had a seizure once every three-four months although she tried various monotherapy and then combination of AEDs, including carbamazepine (CBZ) 600 mg per day, sodium valproate (VPA) 800 mg per day, VPA 800 mg combined with topiramate 100 mg per day, VPA 800 mg combined with lamotrigine (LTG) 100 mg and CBZ 600 mg per day, and finally oxcarbazepine (OXC) 900 mg per day, LTG 100 mg per day and clonazepam 0.5 mg per night before visit to our hospital. Presurgical evaluation of refractory epilepsy was advised several times at the local hospital, unfortunately, the patient denied because of poor economic condition. She noted specific triggers: noisy environment, emotional excitement and fatigue. She stopped AEDs intermittently over the past 20 years, without any change of attack frequency. She had been seizure free for 6 months prior to the event that caused her to come to the ER.

**Fig. 1:**
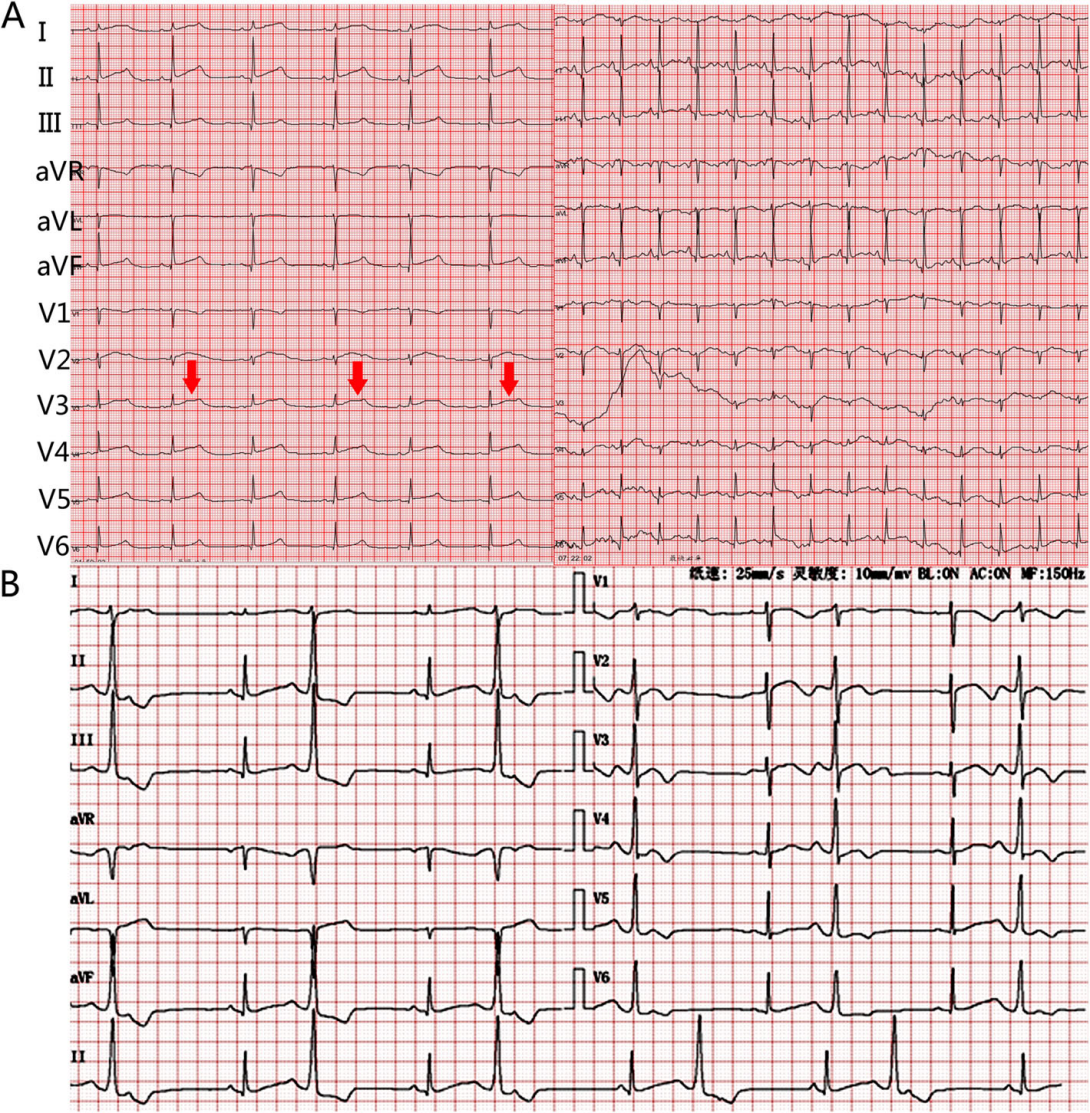
Electrocardiogram of the patient. **A** 24 h dynamic electrocardiogram (Holter) of the patient (25 mm/s, 10 mm/mV). Average heart rate 64 bpm (range 47-109 bpm), corrected QT (QTc) interval of 470 ms (maximum 595 ms). Typical biphasic and notched T wave was presented and marked by red arrow. **B** Emergent electrocardiogram during an attack on the second evening after admission (25 mm/s, 10 mm/mV). Average heart rate 66 bpm, QTc interval of 470 ms. Frequent ventricular premature beats were also recorded

Her general exam was essentially normal with heart rate 72 beats per minute (bpm). Neurological physical exam was essentially normal except for slightly weakened muscle strength diffusely in ER. However, another three attacks were observed soon and on continuous cardiac monitor; they were noted to be accompanied by paroxysmal ventricular tachycardia with frequent ventricular premature beats (bigeminy coupled rhythm). After being treated with IV midazolam and IV amiodarone, the patient was diagnosed epilepsy and arrhythmia and was sent to the inpatient ward. Laboratory data confirmed normal complete blood counts, urinalysis, liver and renal function, glucose, electrolytes, myocardial infarction markers and coagulation functions. Head CT and 24-h VEEG monitoring were both unremarkable. 12-channel dynamic ECG showed a markedly prolonged corrected QT interval (Fig. 1A). Several possibilities were suspected: 1) chronic refractory epilepsy caused cardiac impairment; 2) the events were related to long term use of AEDs with cardiac side effects, such as CBZ, OXC; 3) she had comorbid cardiac disease. We stopped OXC and sought cardiology consultation.

**Fig. 2:**
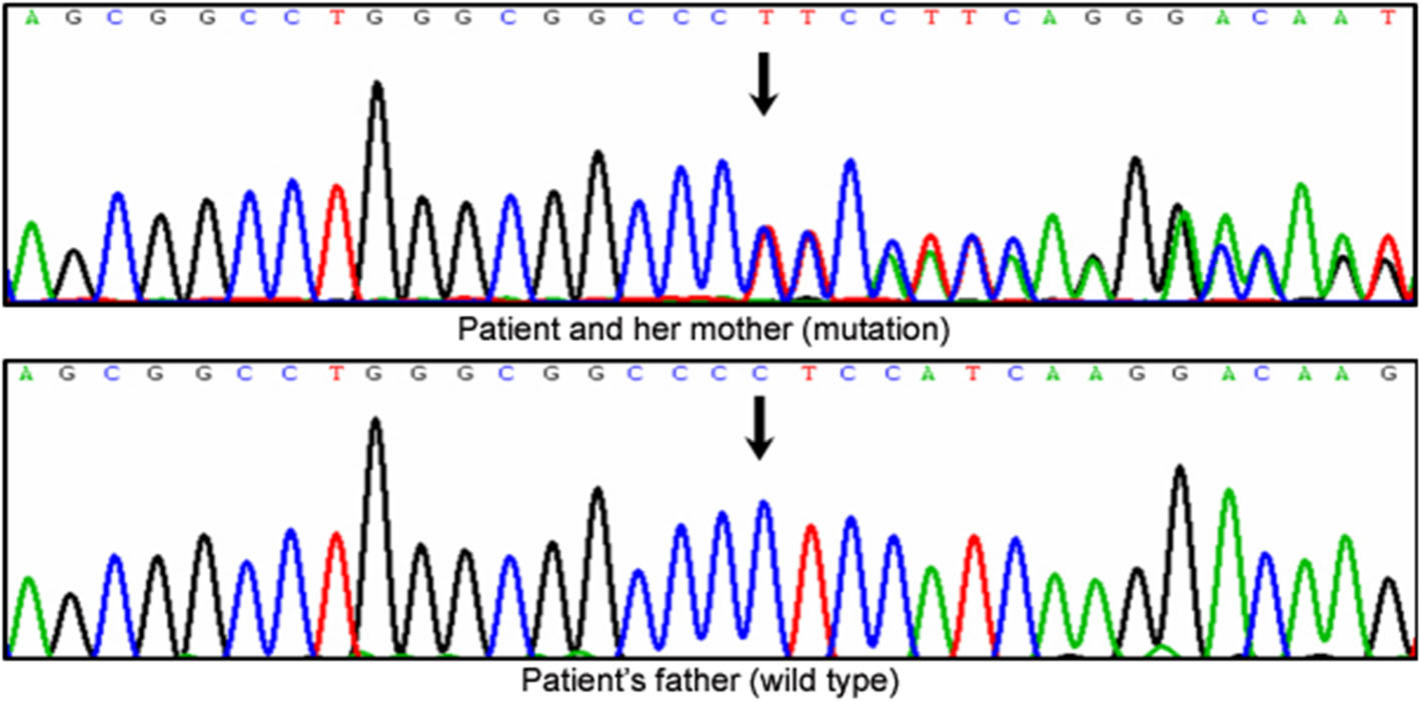
Electropherogram. DNA forward sequence of the *KCNH2* gene showing a *c*.1815delC heterozygous mutation causing a *p*.Ser606fs frame shift and the premature termination of the protein encoded by this gene. The patient and her mother carried the mutation whereas her father had wild type at this point

On the second evening after admission, the patient became irritable suddenly, and complained of an acute tight pressure in her chest accompanied by palpitations. Emergent bedside ECG revealed frequent ventricular premature beat and prolonged QT interval (Fig. 1B). 5% sodium bicarbonate was given intravenously. One hour later, the ECG monitor showed ventricular tachycardia with heart rate of 220 bpm. We gave cardio-pulmonary resuscitation and the sinus rhythm recovered 2 min later. Meanwhile, the patient’s husband noticed that his wife’s seizure was frequently triggered by the ringing of a cellphone belonging to the family member of another patient in the same room, and asked that we move her to a quiet room. This is an important clue because epileptic seizures triggered by noises are rare. Though there are reported syndromes such as autosomal dominant partial epilepsy with auditory features (ADPEAF), including cases with seizures provoked by auditory stimuli, [8] the most prominent feature is seizures accompanied by auditory symptoms (seen in 55–100% of five ADPEAF families caused by LGI1 mutation) rather than seizures provoked by auditory stimuli [9]; in fact, seizures in 82% of familial ADPEAF were unprovoked) [10]. This trigger, combined with the paroxysmal ventricular tachycardia and ECG, made us suspect the diagnosis of LQT2, in which 60% of the events were trigged by acoustic stimuli (e.g. a sharp voice) [11]. The cardiovascular specialists also diagnosed LQTS and advised an implantable cardioverter defibrillator (ICD) and exome sequencing, which confirmed LQT2 by the identification of a *c*.1815delC heterozygous mutation in *KCNH2*, which causes a *p*.Ser606fs frame shift and the premature termination of the encoded protein (Fig. 2). The patient refused ICD implantation because of bad economic state. β blocker and oral potassium was given and guidance on lifestyle modifications including avoiding QT-prolonging drugs, strenuous exercise, exposure to abrupt acoustic stimuli was advised based on expert consensus recommendations [12]. Diagnosis of epilepsy was ruled out because of her atypical trigger factors and aura before attack, along with normal 24-h VEEG monitoring, therefore, all AEDs were withdrawn. She was discharged with oral β blocker and potassium after having no further episodes for 1 week.

The patient reported no family history of epilepsy, cardiac disease, sudden death or psychiatric disease. However, her mother was also confirmed to carry the mutation but refused further evaluation. No mutation was detected in her father (Fig. 2). We followed the patient biannually for 5 years and she reported rare attacks (1–2 per year) with incentives: sharp ringtone, hunger, climbing stairs and staying up.

## Discussion and conclusions

Our case illustrates how long LQTS can masquerade convincingly as epilepsy, leading to delay in diagnosis and mistreatment with AEDs, exposing the patient to a high risk of sudden cardiac death. It raises the importance of cardiac evaluation in epilepsy patients to rule out cardiac diseases and to steer therapy away from AEDs with cardiac side effects. The National Institute for Clinical Excellence (NICE) guidelines for the investigation of first seizure recommend screening ECG [13]. Reevaluation of the diagnosis should be reconsidered in refractory epilepsy patients. Misdiagnosis is the primary cause of pseudoresistant epilepsy, especially misdiagnosed cardiac arrhythmia, which is self-limited, recurrent and easily interpreted as epileptic seizures. Refractory epilepsy cases with atypical features such as an abnormal feeling in the chest before an attack, brief unconsciousness and pallor before convulsive movements, and rapidly return to baseline alertness should consider careful cardiac evaluation.

Early recognition of LQTS is essential for early intervention and significant reduction of mortality. However, delayed diagnosis was commonly seen in 39% of a New Zealand LQTS cohort and the most frequent misdiagnosis was seizure disorder [1]. Additionally, an initial diagnosis of seizure disorder was associated with a longer diagnostic delay of LQTS than were other misdiagnoses, [1] with median 11.8 years (range 9.5–23 years), comparing with only 1 year (range 0.2–15) with other diagnoses, and 4 patient even occurred sudden unexplained death [1]. The diagnostic delay of our case was almost as the longest (23 years) in this study [1]. In another study of familial long QT syndrome in Australia, 9% experienced a delayed diagnosis for at least 12 months and 68.8% of them were initially misdiagnosed, similar to our case, 72.7% of the misdiagnosed cases were diagnosed as epilepsy [14]. Those clinical evidence highlights and reinforces the complexity of clinical diagnosis of LQTS and the need to maintain highly suspicion for cardiac etiology even in patients with a longstanding diagnosis of epilepsy.

Except for clinical characteristics, diagnosis of LQTS depends on prolonged corrected QT (QTc) interval (over 0.44 s calculated by Bazzett’s formula) on ECG. Sometimes, seizures might be the only presentation of LQTS without apparent ECG abnormalities. A resting standard ECG presents with QTc in normal zone was seen in 25–50% of genetically proven LQTS patients because of combination of variable penetrance, the effect of modifying genes and individual variability in QT duration [15, 16]. Diagnostic delay may be caused by overreliance on automatic calculation of the QTc interval provided by digital ECG machines, which may overlook the prolongation of the QTc interval when ECG changes are subtle. Therefore, careful manual analysis of the QTc should be practiced as a routine clinical skill. Exercise and sports can prolong the QT interval and an exercise test may be helpful for those patients suspected LQTS, who show a normal QT interval on resting ECG. Additionally, because of the QTc is a dynamic parameter influencing by a variety of cellular and environmental factors, repeated ECG and especially 24-h ECG Holter recording is also useful for uncovering the dynamic changes in the QTc in congenital LQTS patients and further differentiate different LQTS subtypes, for example, Page et al’s study indicated a different pattern of QTc prolongation in LQT1 and LQT2 patients during 24-h Holter period. LQT1 typically have adverse cardiac events during high sympathetic tone and more likely to have diagnostic QTc prolongation during the day time hours, and on the contrary, LQT2 patients show QTc prolongation during night time hours, especially 3–4 AM [17]. The conclusion of this study explained the rationality of normal ECG presented at the first attack of our patient who is LQT2 subtype, was more likely to present normal QTc in resting standard ECG which is performed during day time. Additionally, auditory stimulus as a typical trigger is also an important diagnostic clue for LQT2, which is also an obvious inducing factor in our case. In patients with provoked seizures precipitated by conditions correlated with adrenergic surge, such as physical exertion, sudden exposure to a noisy environment, fatigue, or emotional agitation, cardiac causes should be highly suspected and exercising or 24-h Holter ECG evaluation should be performed even resting standard ECG is normal.

The main mechanism of LQTS is slowing of the outward potassium current or slowing of the inactivation of the inward sodium current during the third phase of the cardiac action potential. Changes in ion flow across the cardiac membrane lead to a longer action potential and QT interval. Potassium ion channel pathology plays an important role in the etiology of both epilepsy and LQTS. Although normal 24-h VEEG excluded epilepsy in our subject, a history of epilepsy (ascertained by EEG and convulsive attacks) was reported more commonly in LQT2 (39%) than other subtypes (10%), [1] and seizure phenotype was identified in ~ 30% of LQT2 patients with pathogenic *KCNH2* gene variants [18]. Anderson et al’s study explored the prevalence of EEG-identified epileptiform activity among patients with LQTS and found epilepsy diagnosed by an epileptologist on the basis of clinical findings and EEG studies were overrepresented in patients with LQT2 comparing with all other LQTS subgroups (3.7% vs. 0.7%, *p* = 0.0126) [19]. Abnormal repolarization of the *KCNH2* encoded Kv11.1 potassium channels in hippocampus and cortex can cause seizures in LQT2 patients [18]. Additionally, the potassium channels play an important role in cerebral potassium homeostasis and buffering, which dysfunction caused by the mutation can result in elevated extraneuronal potassium level (is epileptogenic itself). However, another possibility is the LQTS patients might carry additional genetic modifiers, which results in epilepsy [20]. If both LQTS and epilepsy were diagnosed, QT-prolonging AEDs should be avoided, mainly sodium channel blockers such as fosphenytoin, CBZ, OXC and LTG.

LQTS are autosomal dominant inherited channelopathies. To date, at least 15 genes and 600 mutations have been reported associated with LQTS [21]. Approximately > 95% of genotype-positive LQTS stem from mutations involving potassium voltage-gated channel subfamily Q member 1 (*KCNQ1*), *KCNH2* and sodium voltage-gated channel alpha subunit 5 (*SCN5A*) [22]. The *KCNH2* mutation in our patient is also autosomal dominant mode. Genotype phenotype correlation exists in LQTS. The different genetic subtypes have specific clinical presentation, ECG abnormalities, prognosis and appropriate management. Arrhythmic episodes with broad T waves on ECG are typically triggered by physical exertion (62%), like swimming, in LQTS type 1 (LQT1), [23] but LQT2 and LQT3 patients tend to have episodes during sleep or at rest, [23] which might be the reason of a high misdiagnosis rate. Again, auditory stimulation, emotional stress and postpartum period are typical triggers for LQT2 events with biphasic or notched T wave [11, 24]. For our patient, diagnosed as LQT2, biphasic and notched T wave was typically presented in the ECG (Fig. 1A), however, her first episode triggered by physical exertion were more common in reported LQT1 (62%) instead of LQT2 (only 13%), but her overall inducing factors including noisy environment and emotional excitement (43%) were similar to reported LQT2 [25]. Her episodes attacked mostly during daytime were partly different from 29% events in LQT2 attack during sleep [25].

For all patients with LQTS, life-style modifications were advised in the AHA/ACC/ESC guidelines for clinical management, [12, 26] such as exposure to abrupt loud noises and competitive sports, which is especially important for our patient because her symptoms has been induced by those factors. Treatment of LQTS includes medicine (β blockers), left cardiac sympathetic denervation, and ICD placement. Although ICD placement is advised based on the class I evidence for survival of a cardiac arrest in recommendations for device-based therapy in LQTS, [12, 27] our patient denied it because of bad economic situation [27]. Other clinical factors that suggest benefit of ICD placement are syncope despite β blocker therapy (class IIa) and high-risk categorization (e.g. diagnosis of LQT2 with sequence variations encoded in the pore region, or LQTS type 3 (LQT3)) (class II b) [27]. β blocker therapy was confirmed to be effective in reducing the risk of cardiac events in genotyped LQTS patients and indicated clinically in LQTS unless a contraindication exists, such as asthma [28]. Long-acting or sustained-release β blockers should be preferred for only once or twice daily administration and stable blood levels, [12] therefore, sustained-release metoprolol succinate 47.5 mg every morning was given to our patient. Additionally, abrupt discontinuation of β blockers might exacerbate the arrhythmia and should be avoided [12].

Our case highlights the need for careful and repeated consideration of the diagnosis and etiology of epilepsy, especially for evaluation of potential cardiovascular disturbances. Especially, for refractory epilepsy cases with atypical features such as feeling of tight pression in the chest before an attack, accompanied by palpitations and brief unconsciousness before convulsive movements, and awareness rapidly returned to baseline; along with cases with provoked seizures precipitated by conditions correlated with adrenergic surge, cardiac causes and LQTS should be highly suspected. Early recognition of LQTS can help physicians avoid mistreatment, especially, the long term use of AEDs with cardiac side effects; and prevent attacks of fatal arrhythmia and sudden death.

## Data Availability

The data that support the findings of this case are available on request from the corresponding author. The data are not publicly available due to privacy or ethical restrictions.

## Abbreviations

ADPEAF: Autosomal dominant partial epilepsy with auditory features
AEDs: Anti-epileptic drugs
bpm: Beats per minute
CBZ: Carbamazepine
CT: Computed tomography
ECG: Electrocardiograph
EEG: Electroencephalograph
ER: Emergency room
ICD: Implantable cardioverter defibrillator
KCNH2: Potassium voltage-gated channel subfamily H member 2 gene
KCNQ1: Potassium voltage-gated channel subfamily Q member 1
LQT1: LQTS type 1
LQT2: LQTS type 2
LQT3: LQTS type 3
LQTS: Long QT syndrome
LTG: Lamotrigine
NICE: National Institute for Clinical Excellence
OXC: Oxcarbazepine
QTc: Prolonged corrected QT
SCN5A: Sodium voltage-gated channel alpha subunit 5
VEEG: Video-EEG
VPA: Sodium valproate
